# Policy challenges of food advertisements from the viewpoints of Stakeholders: A qualitative study

**DOI:** 10.1002/fsn3.1482

**Published:** 2020-03-11

**Authors:** Fatemeh Mohammadi‐Nasrabadi, Yeganeh Salmani, Seyed Mohsen Banihashemi, Arezoo Haghighian Roudsari, Azizollaah Zargaraan, Fatemeh Esfarjani

**Affiliations:** ^1^ Research Department of Food and Nutrition Policy and Planning Faculty of Nutrition and Food Technology National Nutrition & Food Technology Research Institute (NNFTRI) Shahid Beheshti University of Medical Sciences Tehran Iran; ^2^ Faculty of Communication and Media Sciences IRIB(Media) University Tehran Iran

**Keywords:** food advertisements, media, policy challenges, stakeholder

## Abstract

This study aims to explore the stakeholders’ views and suggestions about the policy challenges of food advertisements. Sixteen semistructured interviews were held with media and the food industry experts. Directed content analysis and constant comparison methods were used to obtained categories until subthemes were extracted, and the results were shared as member checking with the stakeholders. Two categories in two themes and seven subthemes were investigated based on the perspectives of the stakeholders: (a) Creating food advertisements and (b) Regulations and rules of food advertising. Few strict guidelines and rules are governing food advertising in Iran. Some factors influencing the general approach in nutrition policy and particularly the choice of policy options and instruments that can be placed in this area, including economic factors, political leadership, lack of political, and systematic monitoring of food advertising status, will were perceived as powerful constraints in advertising policy. Strong links must be established between all sectors that have a bearing on healthy food (the media, public health community, food industry, and consumers). The research findings seek to offer policy options for both the government and the stakeholders for challenging future policies of food advertising.

## Graphical abstract

Watching TV is significantly associated with increased consumption of unhealthy foods, including fast foods, and there is sufficient evidence that TV advertising influences food preferences, purchasing requests, and eating behavior. Unhealthy dietary habits, for example, switching from healthy traditional foods to fast foods, which contain high amounts of fat and sodium and low fiber are the key driver behind developing noncommunicable diseases. As there is a relationship between food advertising and food choices, by modifying people's habits and food choices.

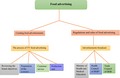

## 1 INTRODUCTION

Advertising in business is a type of communication that persuades and encourages people to take a particular action. Advertisements can be a tool for the introduction of various foods and services to the public. The effects of TV food advertisements have been the issue of extensive research over recent decades. According to the systematic reviews of evidence, television (TV) advertising is responsible for a large share of the marketing of unhealthy foods (World Health Organization, 2011). Watching TV is significantly associated with increased consumption of unhealthy foods, including fast foods, and there is sufficient evidence that TV advertising influences food preferences, purchasing requests, and eating behavior (Aktaş Arnas, 2006; Chang & Nayga, 2009). A systematic review and meta‐analysis study in 2019 showed that most self‐reported studies examined the effect of food advertising on consumer shopping behavior (Qutteina, De Backer, & Smits, 2019). In order to battle the spread of unreliable information, it will be necessary for food risk stakeholders to actively engage with users to correct any sophistries (Rutsaert et al., 2013).

The Islamic Republic of Iran Broadcasting (IRIB) is the head body responsible for all media communications, including advertising through media in Iran. The Ministry of Health and Medical Education, as the main body responsible for food safety in Iran, introduced new regulations describing the laws prohibiting advertising and the introduction of unauthorized and unhealthy foods and services in the internal and international mass media (Zargaraan, Dinarvand, & Hosseini, 2017).

Obesity is among the leading causes of elevated cardiovascular disease (CVDs) mortality and morbidity. Overweight and obesity are among the most important emerging health issues of our time (Akil & Ahmad, 2011). A survey in 31 provinces of Iran in 2011 indicated that the weighted prevalence of overweight and obesity was 34.5% and 21.5%, respectively (Kolahi, Moghisi, & Ekhtiari, 2018). Unhealthy dietary habits, for example, switching from healthy traditional foods to fast foods, which contain high amounts of fat and sodium and low fiber, are the key driver behind developing noncommunicable diseases. As there is a relationship between food advertising and food choices, by modifying people's habits and food choices (Reisch et al., 2013).

It is also important to consider the potential importance of consumers' understanding of advertising and nutrition (Gunter, 2016).

Thus, to the best of our knowledge, this is the first study to explore policy challenges for food advertisements based on stakeholders' views; it further seeks to offer policy options to reduce the challenging future strategies of food advertising to help community health.

## 2 MATERIALS AND METHODS

The qualitative data are considered useful in explaining the concepts and other descriptions of the phenomenon at hand, which suited our context of the study (Berg, Lune, & Lune, 2004). There are different techniques to collect data qualitatively; however, observations, interviews, focus groups, and documents have been considered pivotal (Myers, 2000).

The qualitative semistructured research was facilitated by two researchers, one led the discussion using open‐ended questioning techniques to elicit participants’ own experiences and views, and ensure all participants had an opportunity to take part and the second audio‐recorded the session, summarized and noted down for group checking and reflection during the session. New ideas and questions are created when the participants discuss to each other. Through the discussions, the perspectives change and develop (Taylor, Bogdan, & Devault, 2015).

### 2.1 Member checking: a tool to enhance trustworthiness

In qualitative inquiry methodology, member checking is used and defined as a quality control process by which a researcher seeks to improve the accuracy, credibility, and validity of what has been recorded during a research interview. Member checking is also known as participant verification, informant feedback, respondent validation, applicability, external validity, and fittingness. The participants check to see whether a “true” or authentic representation was made of what he or she conveyed during the interview. Member checks may include sharing all of the results with the participants and allowing them to critically analyze the findings and comment on them. The most significant benefit of conducting member checks is that it lets the researcher the opportunity to verify the accuracy and completeness of the results, which then helps to improve the validity of the study (Drury, Francis, & Chapman, 2007; Harper & Cole, 2012).

### 2.2 Subjects

In this qualitative study, semistructured, interviews were held with 16 media and food industry experts in 2019.

The samples were selected via purposive sampling and snowballing. Participants were considered eligible if they were acknowledged experts in the food industry and also specialists in the food advertisements. The interviews were held until data saturation was reached (Guest, Bunce, & Johnson, 2006). The questions of open‐ended interviews were used to identify the policy issues of food advertisements. The experts’ voices were recorded, fully transcribed, anonymized, and checked for accuracy to obtain categories until generating themes using directed content analysis and constant comparison methods (Pope & Mays, 2013).

### 2.3 Sample selection

The 16 stakeholders were recognized from the governmental, private sectors, and nongovernmental organizations (NGO) and included: (a) a research member of media University, (b) member of the IRIB Policy Council (two person), (c) advertising manager and planner (four person), (d) film producers who make teasers for advertising companies (two person), (e) a communication and advertising consultant, (f) a secretary of Health Policy Council for IRIB, (g) a deputy of Food and Drug Administration, (h) a secretary of the Vegetable Oil Association as a subset of Federation of Iranian Food Associations, (i) Head of networks (two person), and (j) a managing director of a food factory.

### 2.4 Data gathering

The questions were then assessed for their content by two experts. Next, they were pretested with two experts from the media and an advertisement company and based on their comments, and few changes were made. The main questions were followed by some probe questions to acquire the required data fully. The interview guide protocol is shown in Table 1.

**Table 1:** Interviews guide protocol

Questions
1. How does advertisement get created? Content By whom (team)
2. In your opinion, is there any overview of the accuracy of the advertisement content? If yes, by whom?
3. In your opinion, are there any laws and regulations in the field of food advertisements? Which organizations compile these regulations by and how? What are the levers of the implementation of these regulations and laws? Are these regulations adequately informed to the stakeholders? In your opinion, which organizations should be responsible for pursuing policy implementation? In your opinion, what are the weaknesses and strengths of these regulations?
4. To what extent do you consider food advertisements regulations? What do you know about the reasons and the barriers to non‐compliance?
5. What is your opinion about food advertisements that are currently playing on TV?
6. Do you think the advertisements provide the correct information to the consumer? What are the positive and negative points of edible oil advertisements?
7. What is your suggestion regarding food advertisements?

The data needed for the study were gathered through semistructured in‐depth interviews. All of the participants signed a written informed consent form before the start of the interview, and the explicit agreement was sought from all of them for audiotaping (Fritz, 2008). The duration of each interview session was 45–55 min. The responses of other participants were recorded only upon their permission. Notes were written whenever recording the interview was not probable.

Furthermore, the interviewees were secured that the collected data would remain private. In order to protect their identity, each participant took a coded number. Each team consisted of one moderator, one observer, and two notetakers. The moderator was a flexible, open‐minded, active listener, able to good communication and establish a report with the participants and encourage them to talk comfortably. She started the interviews by presenting the objectives of the study and provided a short orientation as a discussion ice breaker. The notetakers were swift and careful in writing. The observer watched what happened but had no active part in the discussions (Krueger, 2009; Morgan, 1993).

At the first of each session, the moderator introduced the survey team and explained the purpose of the study. The samples were encouraged to share their views freely with the moderator.

#### 2.4.1 Validity and reliability

To assure data accuracy and consistent comments, the validity and reliability issues should be concerned by researchers during planning a study, analyzing findings, and judging the quality of the study. For the present study, triangulation was done by collecting data through the convergence of information from different types of participants in the media and food industry. The findings were examined and confirmed by some of the key informants who met the inclusion criteria but did not participate in the research. To confirm dependability, three researchers controlled again. Then to help improve the accuracy, credibility, validity, and transferability, the results were shared and were summarized after discussions, and also, the policy documents of food advertising were reviewed in the meeting for member checking with the experts in all sectors.

### 2.5 Data analysis

The transcription of each interview audio record was compared with the notes to fix potential discrepancies. Transcripts were examined exactly basis with codes being assigned to segments of the data by highlighting the exact words from the text that appeared to capture critical thoughts to provide initial coding frame developed. The process of final constant comparison allowed for the coding frame to be modified as analysis developed.

The team members read line by line the text independently to derive codes in order to capture key concepts. Based on the similarities, the codes were classified. To determine and group the codes into meaningful themes, the emerged categories were used. Also, the constant comparative technique was used to determine emerging themes (Charmaz, 2006).

## 3 RESULTS

The results conducted that the majority of the interviewed stockholders (89%) were in the age range of 40–50 years, and their education level was master and higher degrees (87%).

Two categories in two themes and seven subthemes were generated based on the views of the participants: 1) Creating food advertisements (advertising agencies), and 2) Regulations and rules of food advertising (Table 2). Then, the results were shared as member checking with the stakeholders.

**Table 2:** Open coding results (essential concepts extracted according to priority)

Categories	Themes	Sub ‐Themes
Creating food advertisements (advertising agencies)	The process of TV food advertising	Reviewing the brand objectives of TV advertising Preparation of the advertising scenario ‐ Brief description of the brand ‐ Product introduction Customer service Production unit
Regulations and rules of food advertising	Advertisements broadcast	The Health Council of IRIB (the content of food advertising) ‐ Compiling regulations concerning the content of advertisements Trade Council of IRIB (standard broadcasting advertisements) ‐ Reviewing the manufacturer's documents ( health license and production license) ‐ A trilateral agreement between the advertising company, trade council, and the manufacturer ‐ Broadcast review board Ministry of Health and Medical Education ‐ Deputy of Health ‐ Food and Drug Administration

### 3.1 1) Creating food advertisements (Advertising agencies)

All stakeholders cited that how does advertisement get created:

**“**Advertising companies play the role of intermediary between producer and TV so that they start the advertisement after receiving the order of the work stages and then make an advertisement for broadcasting it on the TV.

After a corporate TV advertising project enters advertising companies, it has to go through four steps to finalize it and appear on the TV. These four steps are:

a) Reviewing the brand objectives of TV advertising

In the beginning, the producer introduces the target of product advertising and overall situation of competitors. Then, according to the goals, whether the targeting is correct and can be achieved through advertisements are examined or, if necessary, make changes. Finally, by reviewing everything, a roadmap is drawn up to continue the route to the completion of the project.

b) Preparation of the advertising scenario

At this stage, according to the information obtained from the previous stage and according to the designed goals set, the unit of creativity and ideology develops an advertising scenario and designing it by unique ideas. Scenario content development and advertising slogan design are some of the things that are being made at this stage.

c) Customer service

The customer service maintains its direct relationship with the customer in all stages of the project and is responsible for tracking the ordering requirements. In this way, it can be assured that the customer's requirements are always taken into account and that a regular work report is provided.

d) Production unit

The production team (director, producer, production manager, animator, graphic artist, etc.) builds a teaser or content tailored to the scenario they design. After reviewing the prepared teaser, they modify it to reach the final approval.”

### 3.2 2) Regulations and rules of food advertising

#### 3.2.1 Advertisements broadcast

Reviewing the history of food advertisements shows that there are few guidelines governing food advertising. Few participants mentioned that: “*Changes in the political situation and ministries hamper the collaboration and development of advertising policy.*”

The majority of the interviewees noted:

“The rules and regulations in the field of food advertising are developed in three major bodies

a) The Health Council of IRIB (Content of food advertising): The majority of the interviewees mentioned that the formulation and notification of regulations, and executive instructions, and standards for the establishment of harmonization and compliance in the production and distribution of programs are, generally, the responsibility of this council.

b) Trade Council of IRIB (Standard broadcasting advertisements): The majority of the interviewees mentioned that to display food advertisements, the basic factory documents are checked by this council, and then the trilateral contract is signed with the advertising company and the manufacturer. After the advertisements are generated, the Review Board will review it and, if it has the required criteria, the broadcast code will be allocated to it.

c) Ministry of Health and Medical Education: Food and Drug Administration annually sends a list of unhealthy products by law and by order of the Minister of Health and Medical Education to IRIB and the advertising agencies. According to this law, unhealthy foods should not be advertised under any circumstances.”

### 3.3 Challenges of Food advertisements derived from stakeholders’ perspectives

- Lack of up‐to‐date rules in the field of food advertising
- Lack of governmental support in the implementation of lows for food advertisements
- The lack of coordination of food advertising legislation among the organizations, institutions, associations, and the Ministry of Health and Medical Education,
- Existence of seductive, and false advertising,
- Lack of control of food advertising scientific claims
- Lack of specialists (nutritionists, pediatrics, psychologists, sociologists, etc.) for continuous monitoring of advertising in Health Council of IRIB and the Broadcast Review Board.

**Figure 1:**
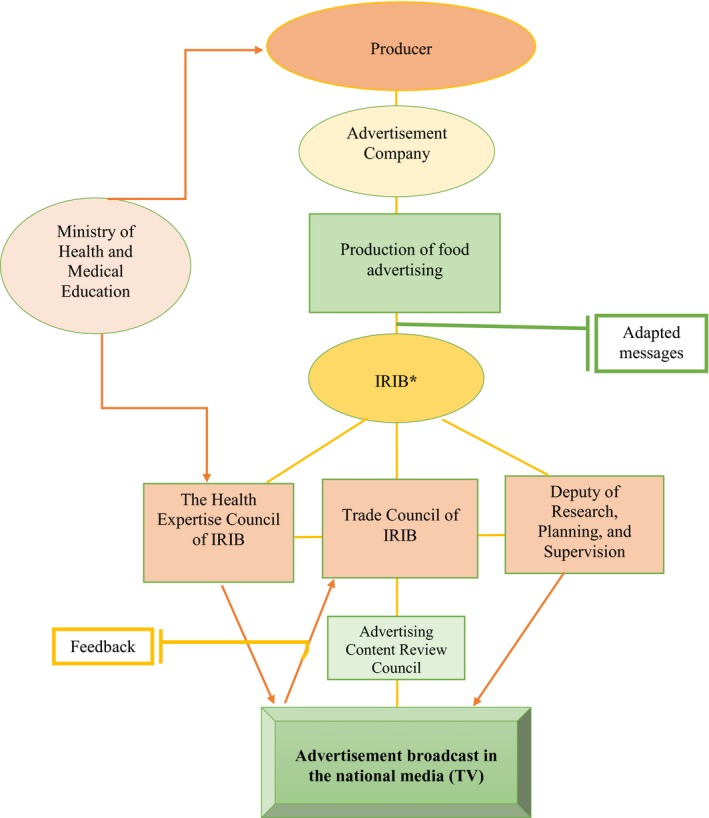
Conceptual framework of food advertisements

Briefly, the results showed that food advertising policy depends on a combination of different factors, operating at several levels (Figure 1).

## 4 DISCUSSION

To our knowledge, this is the first study providing policy challenges of food advertisements from the viewpoints of stakeholders.

Advertising that misleads health consumers may promote unnecessary and inappropriate engagement in health services and may therefore negatively affect consumers’ ability to exercise autonomous decisions relating to their care (Holden, 2019).

A television advertisement is a form of advertising that promotes products, services, organizations, or ideas that sometimes become part of our cultural vocabulary and receive a lasting place in the community's mind (Vaynerchuck, 2011). There can be hundreds of steps that go into producing TV advertisements, but some of the most crucial aspects take place long before the commercial is filmed. A concept is planned, scripted, and then explained on storyboards that detail how the advertisement will progress. After the storyboard is approved, actors or other talents will be hired, depending on whether the commercial includes live‐action or animation (Sirotin, 2013).

After the advertisement is shot, it will go to postproduction, where it will be edited and readied for distribution. At every point in the process, there is potential for an idea or its execution to fall flat. Producing a TV commercial requires tremendous attention to detail, no matter what the stage (Graf, 2012). Overall, the findings of these contents are similar to our results, which conducted the process of creating food advertising.

The results of this study indicated that some of the restrictions on food advertising were not implemented. Also, the evidence of a study in the UK showed that the excellent adherence to the limitations recommend that scheduling restrictions on television advertising of unhealthy foods can be efficient in decreasing broadcast of these advertisements—but only in the broadcast slots to which they implement. Without wide‐ranging consideration of all broadcasting, such regulations run the risk of only shifting, rather than reducing the problem. Despite that evidence, the restrictions did not achieve their aim and this is likely to be because they only applied to a very small proportion of all television broadcasts (Adams, Tyrrell, Adamson, & White, 2012).

Organizations such as the Coalition on Food Advertising to Children (CFAC) and the WHO argue that more stringent restrictions for food advertising are essential to address the mounting crisis of obesity as exposure to junk or fast food advertising contributes to an atmosphere that encourages unhealthy foods. Others argue that there is not much evidence to show a relationship between advertising and obesity, and therefore, advertising limitations are not a suitable means to address obesity. Even though the causes of obesity are multi‐factorial, a decrease in food advertising through media is one vital approach for encouraging to make the right food choices (Oommen & Anderson, 2008). Data from a systematic review and meta‐analysis study of the effects of acute exposure to unhealthy food and nonalcoholic beverage advertising on intake in children and adults support public health policy action that seeks to reduce children's exposure to unhealthy food advertising (Boyland et al., 2016).

A systematic review study suggested that exposure to alcohol advertising in the media increases the likelihood of later alcohol consumption (Smith & Foxcroft, 2009). These results have also been seen in relation to food advertising, a study, in the UK, showed that food advertising influences on dietary public health policymaking seem to be somewhat greater than the influence of public health stakeholders in the initial structure of the consultation, and this imbalance may have contributed to the ultimately compromised regulation (Razavi, Adams, & White, 2019).

The outcomes of the present study highlight the ongoing need for new laws regarding food advertisements. Our results are similar to the study in Ireland that indicated the new laws should be passed for controlling advertisements of unhealthy foodstuff broadcasted during programs (Scully et al., 2015). Another study in the UK in 2008 showed that, despite regulation, children are exposed to TV advertising for unhealthy food items. There remains scope to strengthen the laws regarding advertising of unhealthy foods around programming popular with children and adults alike, where current regulations do not apply. Ongoing, systematic monitoring is the requirement for evaluation of the effectiveness of rules designed (Boyland, Harrold, Kirkham, & Halford, 2011).

In a research carried out specific regulations for food advertising in Latin‐American countries, however, the results of studies conducted that, recently, in these countries, governments have not implemented those rules (Bacardí‐Gascón & Jimenez‐Cruz, 2015).

A review study showed that the Ministers of Health at the Pan‐American Health Organization summit in 2014 approved a 5‐year plan to reduce the exposure to unhealthy foods, including sweetened drinks, but no real effort to implement or to sanction those industries, which are violating the legislation has been seen (OPS. Comité Regional de la OMS para las Américas Washington).

The WHO has established regulation norms that include the reduction of exposure to foods with an unhealthy amount of fat, saturated fat, transfatty acid, sugar, and salt. It has also recommended continual monitoring to quantify the number of exposures and strategies of those foods used (Hawkes & World Health Organization, 2007).

Challenges for food and communication‐related research, food marketers, food policy‐makers, and public health authorities require further attention and investigation (Rutsaert et al., 2013).

*Economic factors,* such as limited budgets due to sanctions and high costs of particular policy instruments also expressed in costs to benefits relation, were noticed as powerful constraints in nutrition policy in all countries. Insufficient resources were the main reason for insufficient monitoring and updating the micronutrient intake or status data in various populations. Limited resources on nutrition policies were connected with governmental official's opinions that they were less significant than other public health problems.

*Political leadership*, especially the type of governing occurring in the country, was perceived as an important component in nutrition policy. Liberalism was mentioned to shape the general character of nutrition policy, mainly in the context of policy instruments preferences (especially the educational programmers/campaigns and voluntary food fortification) and it was indicated in most of the countries, like in the north (NL, DK, NO, and EN), central (CZ), and south (ES) of Europe (Jeruszka‐Bielak et al., 2015).

### 4.1 Optimal policy suggestions in food advertisements

**Figure 2:**
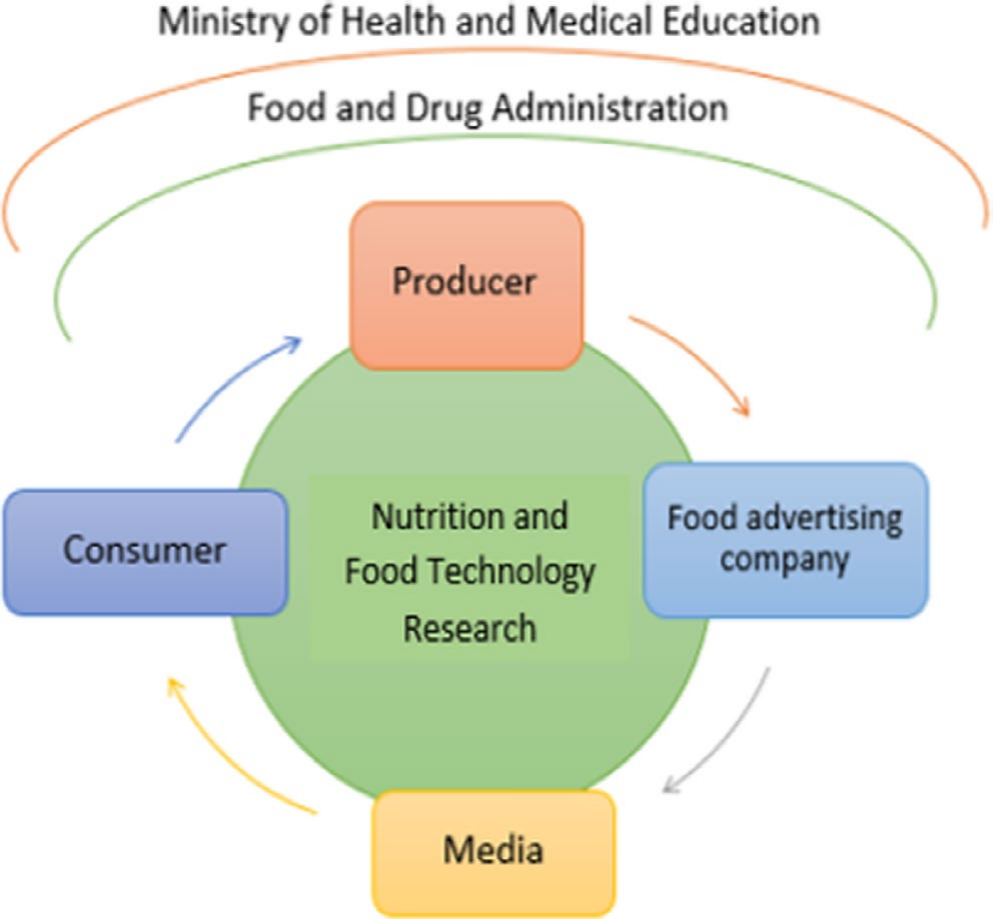
Proposed trend for promoting healthy food advertising

- Determining promotional tariffs for unhealthy foods advertising
- The continues presence of an expert team in the preparation and reviewing of advertising content before broadcasting
- Mentioning the prohibition of advertising in production licenses for harmful foods
- Controlling food claims through conducting daily tests by the Food and Drug Administration
- Increasing the consumer awareness of healthy eating through launching a healthy nutrition advertising campaign as social responsibility
- Updating rules and guidelines for advertising healthy food
- Encouraging entrepreneurs to establish healthy food factories (tax reduction, fanatical supports)
- Optimal use of the empty capacity of factories to produce healthy foods
- Controlling the licensing of manufacturing facilities for the production of harmful foods
- Linking between the Nutrition and Food Technology Research Institute and all sectors to promote a healthy food. (Figure 2).

The Ministry of Health and Medical Education should develop a better‐coordinated mechanism for the food industry and media. On the other hand, all sectors should follow and implement the guidelines and regulations and also consider public health and healthy nutrition as its utmost priorities.

We accept that policies are formulated and implemented within specific historical contexts, and outcomes are dependent on time and place. However, this does not mean that nothing can be done to change policy. We suggest that the current crisis in health demands rigorous and comprehensive analysis of the policy process and its influence on policy effectiveness, as input into future policymaking.

Even though different policies are put in place across many countries about food advertising, governments will still tend to regard obesity not as a major health problem but as a tendency shown to food by an individual. It is also recommended that criteria be proposed for monitoring and controlling food advertisements. This perception ought to transform in order to reduce noncommunicable diseases, which is already affecting the future generation.

### 4.2 Limitations

Limitations of this review mainly relate to the scope that includes the restrictions to TV advertising and not other media (e.g., websites, text messaging, sporting events, product packaging, and billboards).

## 5 CONCLUSION

Stakeholders in all related sectors should be sharing common goals and responsibilities, and strong links must be established between them to promote the health of the community.

The food industry and advertisers should work more jointly with the government and health promoters to develop a mutually agreed‐upon set of standards by which advertising will be regulated. The research findings attempt to offer policy options for both the government and the stakeholders for healthy food advertising to prevent changing the food pattern and reduce noncommunicable diseases. These results could be used for designing appropriate strategies to increase the quality of food advertising through media to make the right and healthy food choices. It is recommended that all sectors face up to their task and further strengthening of the rules is needed.

## CONFLICTS OF INTEREST

All authors declared no personal or financial conflicts of interest.

## ETHICAL STANDARDS DISCLOSURE

Manuscripts describing research involving human participants must include the following statement: "This study was conducted according to the guidelines laid down in the Declaration of Helsinki and all procedures involving research study participants were approved by the [Research Council of National Nutrition and Food Technology Research Institute]. Written informed consent was obtained from all stockholders." All of the stakeholders signed a written informed consent form before the beginning of the interview, and explicit permission was sought from all of them for audiotaping.

## ETHICAL CONSIDERATION

Ethical issues (including informed consent, plagiarism, misconduct, data fabrication and falsification, double publication and/or submission, redundancy) have been totally considered by the authors.
